# Interpretation of Cerebral Oxygenation Changes in the Preterm Infant

**DOI:** 10.3390/children5070094

**Published:** 2018-07-09

**Authors:** Aisling A. Garvey, Elisabeth M. W. Kooi, Aisling Smith, Eugene M. Dempsey

**Affiliations:** 1 Department of Paediatrics and Child Health, Neonatal Intensive Care Unit, University College Cork, T12 YE02 Cork, Ireland; aisling.garvey@ucc.ie (A.A.G.); e.kooi@umcg.nl (E.M.W.K.); smithai@tcd.ie (A.S.); 2 INFANT, Irish Centre for Fetal and Neonatal Translational research, University College Cork, T12 YE02 Cork, Ireland; 3 Division of Neonatology, Beatrix Children’s Hospital, University Medical Center Groningen, University of Groningen, 9713 GZ Groningen, The Netherlands

**Keywords:** near-infrared spectroscopy, NIRS, cerebral oxygenation, end-organ tissue oxygenation, neonate, preterm, individualised patient care

## Abstract

Near-infrared spectroscopy (NIRS) allows for continuous, non-invasive monitoring of end-organ tissue oxygenation. The use of NIRS, cerebral NIRS (cNIRS) in particular, in neonatal care has increased significantly over the last few years. This dynamic monitoring technique provides real-time information on the cerebral and haemodynamic status of the neonate and has the potential to serve as an important adjunct to patient care with some centres routinely utilising cNIRS to aid decision-making at the bedside. cNIRS values may be influenced by many variables, including cardiac, respiratory and metabolic parameters, and therefore it is essential to understand the pathophysiology behind alterations in cNIRS values. Correct interpretation is required to direct appropriate patient-specific interventions. This article aims to assist clinicians in deciphering cNIRS values by providing an overview of potential causes of fluctuations in cNIRS values, illustrated by common clinical scenarios, with particular emphasis on the preterm infant.

## 1. Introduction

Although introduced into clinical care almost 40 years ago, it is only in the last decade that near-infrared spectroscopy (NIRS) has grown in popularity in the Neonatal Unit (NNU) [1]. NIRS provides a continuous, non-invasive measurement of end-organ tissue oxygenation (rSO_2_) and its ease of application and its potential usefulness has contributed to its increasing use, especially in the very preterm infant. Cerebral NIRS (cNIRS) provides a potential window into the cerebral and haemodynamic status of the neonate. The merits of NIRS monitoring have been discussed previously [2] and NIRS monitoring either alone, or in conjunction with other modalities, potentially has an important role to play in care of the newborn. Some centres now routinely use cNIRS to assist in decision-making at the bedside, especially in the care of the preterm infant.

Unlike pulse oximetry which measures arterial oxygen saturation, NIRS measures tissue oxygen saturation, which consists of a combination of arterial, venous, and capillary blood. While NIRS monitoring provides relative regional saturations and reference guidelines have been suggested [3,4], studies to date are heterogeneous in their study population and outcomes [5] and values have also been shown to vary with gestational age (6). There are a number of different devices used with a variety of algorithms incorporated, which may account for some of the variability that exists [6,7,8,9]. In addition, the type of sensor used can produce differences of up to 14% in values [6] and reapplication of the same probe in the same region can result in differences of up to 6% [8].

As clinicians, we are becoming increasingly aware of the importance of interpreting physiological data in the context of each individual infant, instead of a “one size fits all” approach, in order to provide optimum and appropriate individualised care. As a result, the challenge facing many clinicians lies in the interpretation of this relatively new bedside device. The trend of the values and signal itself are rich in information [10,11] but unlike peripheral oxygen saturation levels (SpO_2_), cerebral tissue oxygen saturation is influenced by many potential variables, including cardiac (blood pressure and cardiac output), respiratory (partial pressure of carbon dioxide and oxygen in arterial blood), and metabolic (glycaemia) parameters. Correct interpretation of the cerebral oxygenation values/trends in light of such variables is essential if appropriate interventions are to be implemented, and changes in cerebral NIRS values should prompt a clinical evaluation of the infant to determine the underlying cause.

**Figure 1:**
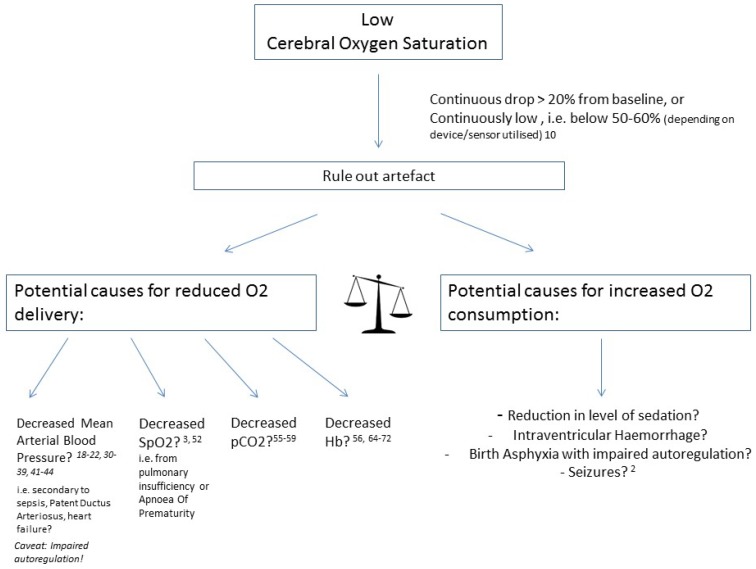
Causes of low cerebral oxygen saturation.

**Figure 2:**
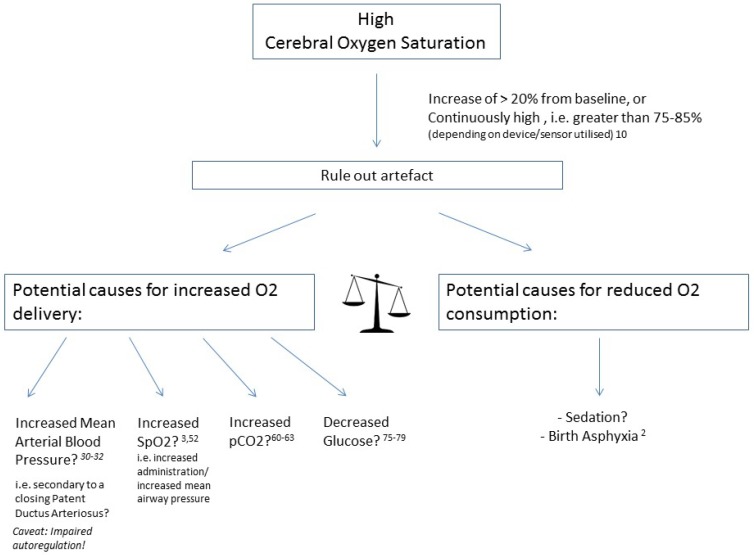
Causes of high cerebral oxygen saturation.

Figure 1 and Figure 2 provide a schematic overview of potential influencing factors of NIRS values.

To gain more insight in the balance between arterial oxygen delivery to the brain and the brain’s oxygen uptake, fractional tissue oxygen extraction (FTOE) has been utilised [12]. In newborn piglets this parameter appeared to correlate well with fractional oxygen extraction (FOE), as measured from blood oxygen content in arterial and venous blood samples. FTOE is calculated by dividing the difference between SpO_2_ and rSO_2_ divided by SpO_2_ ((SpO_2_–rSO_2_)/SpO_2_). This way, FTOE represents the fraction of the delivered oxygen that has been extracted by the tissue measured [12]. In reality, this also partly compensates for low arterial oxygen levels, as is often the case in preterm infants with lung disease, or in infants with congenital heart defects [13]. In these situations, FTOE may be a better parameter for brain perfusion (assuming a relative constant brain metabolism rate) than rSO_2_, and potentially a better measure for the assessment of autoregulation when combined with blood pressure [14].

In this article, we concentrate solely on cerebral oxygen saturation values and suggest an approach to the interpretation of cNIRS at the bedside and provide examples of commonly encountered scenarios in the neonatal unit, with particular emphasis on the preterm infant in the first 72 h of life.

## 2. Hypotension

For over four decades, the question of low blood pressure and the need for use of inotropes has largely remained unanswered. Much of this uncertainty results from a lack of good quality evidence from large randomised controlled trials [15,16]. The association between hypotension and brain injury or poor neurodevelopmental outcome remains controversial [17,18,19,20]. Recently, focus has shifted towards incorporating the use of surrogate markers of end organ perfusion as a means to determine when treatment should be instigated. cNIRS as a surrogate of cerebral blood flow (assuming a stable cerebral oxygen consumption) has gained momentum and has also been utilised to assess inotrope use in preterm infants and their effect on cerebral perfusion and blood flow [21,22,23]. Also, when combined with blood pressure, as a surrogate for cerebral perfusion pressure it has the potential to provide a dynamic, continuous measurement of cerebral autoregulation [24].

Cerebral autoregulation is the mechanism by which cerebral blood flow remains constant despite fluctuations in cerebral perfusion pressure. Preterm infants are at an increased risk of impaired autoregulation, primarily due to the immaturity of smooth muscle cells of their cerebral arteries [25,26]. As mentioned, cNIRS can be used as an assessment of cerebral blood flow, and when combined with blood pressure as a surrogate for cerebral perfusion pressure, the cerebrovascular capacity in an infant can be evaluated. Uncertainty remains, however, regarding the interpretation of these values and the optimum algorithm to validly assess cerebrovascular autoregulation [24]. Accurate identification of periods of impaired autoregulation and cerebral compromise may provide opportunities for appropriate treatment to be initiated and continuously evaluated [27].

**Figure 3:**
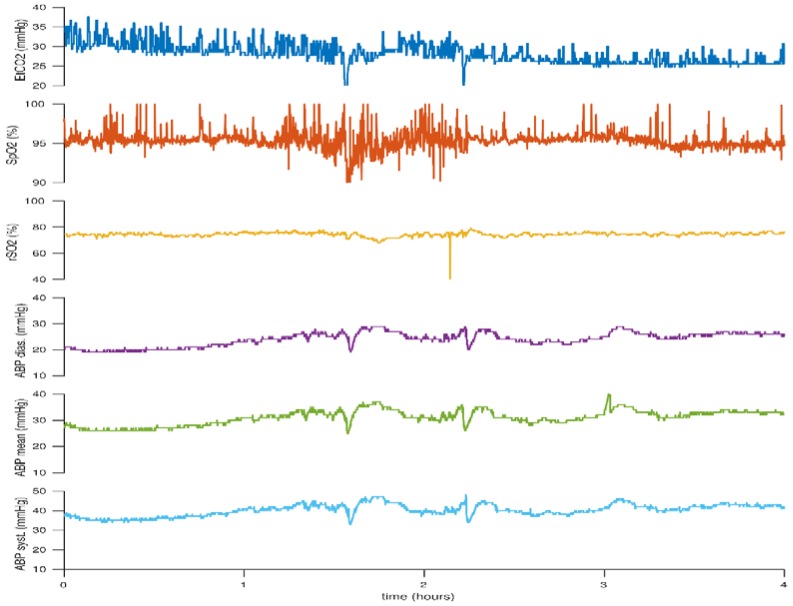
Cerebral rSO_2_ remains stable despite changes in blood pressure. This figure reflects the case of neonate born at 24 + 5 weeks gestation. Birth weight (BW) 660 g, day of life (DOL) 1.

Below we illustrate the advantage of cNIRS when assessing the potential need for intervention in periods of low blood pressure. The figures provided are recorded using a Moberg CNS device (Moberg, PA, USA) and an INVOS 5100 (Somanetics, Troy, MI, USA) NIRS monitor with a neonatal probe. Figure 3 illustrates fluctuations in the infant’s mean arterial blood pressure, with associated periods of low blood pressure, but the cerebral rSO_2_ values remain stable and mean blood pressure spontaneously increases with time. The stable cNIRS trend during this period of low blood pressure may be a useful adjunct in decision-making about interventions, such as whether inotropes should be commenced or not.

**Figure 4:**
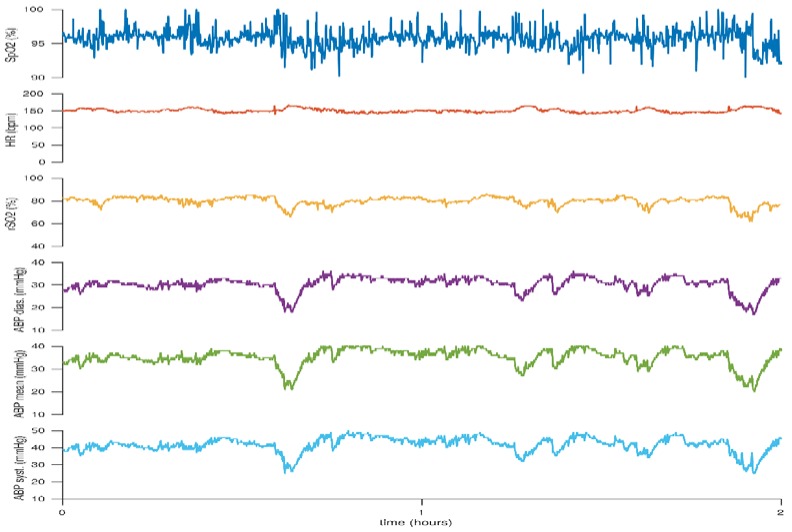
Decreases in cerebral rSO_2_ in association with decreases in blood pressure. This figure reflects the case of a neonate born at 27 + 6 weeks gestation. BW 770 g, DOL 1.

In Figure 4 however, the periods of low blood pressure are associated with periods of simultaneous changes in cerebral oxygenation. This would suggest that cerebral autoregulation is not maintained and this infant may benefit from methods to stabilize the mean arterial blood pressure, although the cerebral oxygen saturation appears to be within adequate ranges.

Low cNIRS values can also provide invaluable information in assessing systemic circulation. A low cerebral rSO_2_ value associated with signs of poor systemic perfusion such as a prolonged capillary refill time (CRT), and increased lactate or poor urine output would probably necessitate treatment to improve cardiac output [28,29] and tissue perfusion rather than an observational approach to the management. However, there is limited evidence from clinical studies to support such an approach.

## 3. Patent Ductus Arteriosus: Significant or Not?

Patent ductus arteriosus (PDA) is the most common cardiovascular finding in preterm infants [30]. Although PDA is associated with significant pathologies, including necrotising enterocolitis (NEC), chronic lung disease, and intraventricular haemorrhage (IVH), causality remains debatable and the optimal management of PDA remains controversial [31]. Numerous studies have sought to provide guidance on what constitutes a haemodynamically “significant” PDA (hsPDA) and how to effectively identify which PDAs require medical/surgical closure [32,33,34]. Although many studies utilise chronic lung disease and ventilator dependence as primary outcomes, Lemmers et al. have demonstrated significantly lower cerebral rSO_2_ values in infants with hsPDAs. In addition, these values normalised to values of infants in the control group following medical closure of the PDA [35,36,37]. This finding was even more pronounced in infants who were born small for gestational age (SGA) [38]. However, other studies report stable cNIRS values despite showing differences in mean arterial blood pressure (MABP) and abdominal rSO_2_ values. It is recognised that with preservation of cerebral autoregulation, perfusion may be maintained in the face of a hsPDA [39,40,41,42,43]. 

An additional potential role of cNIRS is the assessment of cerebral perfusion during medical and surgical therapy directed at hsPDA closure. Ibuprofen has been the first-line medical treatment of PDA for over a decade. Prior to its introduction into routine clinical practice, Patel et al. conducted a randomised controlled trial comparing the effects of indomethacin and ibuprofen on cerebral haemodynamics. Indomethacin resulted in a significant decrease in cerebral blood flow and oxygen delivery after administration, whereas ibuprofen had no negative effects on cerebral haemodynamics [44]. A 2018 study reported that paracetamol administration did not affect cerebral haemodynamics as measured by NIRS. This evidence is useful in supporting the safety profile of paracetamol, which is increasingly prescribed following failure of PDA closure with ibuprofen [45].

The effect of surgical PDA ligation on cerebral oxygenation remains controversial. Huning et al. utilised cNIRS to report that there is no change in cerebral oxygenation during PDA ligation [46]. Contrary to this, studies have demonstrated a reduction in cNIRS values during and immediately after surgical PDA ligation [47,48,49]. Whether this is as a result of impaired blood flow or increased tissue oxygen utilisation during surgery is unclear, with studies reporting conflicting results. Another consideration is that infants who undergo surgical PDA ligation tend to be of greater postnatal age and may have already failed medical/conservative management, which may have an impact on their baseline pre-surgery rSO_2_ values and subsequent outcomes [50].

Studies are now attempting to link cNIRS findings to subsequent neurological outcome. Verhagen et al. have demonstrated a correlation between cNIRS values in the first two weeks of life and neurodevelopmental outcome at 2–3 years [5]. Further to this, Lemmers et al. have noted a relationship between longstanding low cerebral oxygenation values on cNIRS in premature infants requiring surgical ligation for hsPDA and decreased cerebellar volume on MRI imaging at term equivalent age. They postulate that low cerebral oxygenation secondary to hsPDA and systemic steal may cause reduced brain volume and myelination, with subsequent consequences on neurodevelopmental outcomes [50].

There are many physiological variables that influence the haemodynamic significance of a PDA including the oxygen carrying capacity of the blood, fluid and respiratory status, shunt severity, and autoregulatory capacity of the infant. Additionally, echocardiographic indices may not correlate directly with impaired cerebral oxygenation [39,41]. Therefore a thorough evaluation of the entire physiological status of the infant is required to determine the haemodynamic significance of a PDA. cNIRS measurements may prove a useful adjunct to the clinical, biochemical, and echocardiographic assessment of shunt significance and whether PDA closure should be pursued medically or surgically for an individual patient. The impact of post ligation syndrome and its management has not been evaluated with cNIRS.

## 4. Peripheral Arterial Oxygen Saturation

**Figure 5:**
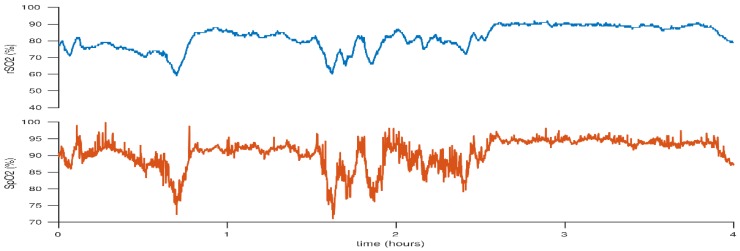
Decrease in cerebral rSO_2_ associated with decrease in peripheral SpO_2_ due to desaturations. The subsequent overshoot is likely due to the increased FiO_2_.This figure reflects the case of a neonate born at 25 + 2 weeks gestation. BW 530 g, DOL 3.

**Figure 6:**
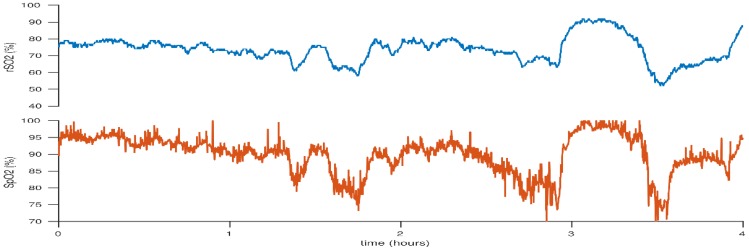
Cerebral hyperoxia following an episode of desaturation treated with increased FiO_2_. This figure reflects the case of a neonate born at 25 + 2 weeks gestation. BW 530 g, DOL 3.

**Figure 7:**
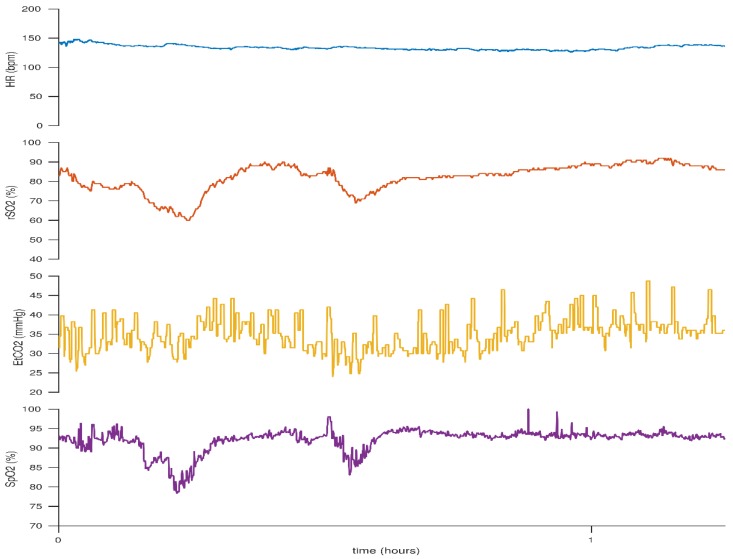
Cerebral rSO_2_ and peripheral SpO_2_ are decreased by reducing administered FiO_2_. This figure reflects the case of a neonate born at 25 + 2 weeks gestation. BW 530 g, DOL 1.

Until recently, pulse oximetry was the sole measure of effective provision of supplemental oxygen to meet metabolic demands. Studies have demonstrated a higher survival rate in infants <28 weeks gestation who were randomised to a SpO_2_ target of 91–95% compared to those in the target SpO_2_ group of 85–89% [51,52]. Clinical trials have also demonstrated an increased risk of morbidity in preterm infants with higher peripheral oxygen saturation targets, specifically an increased risk of chronic lung disease and retinopathy of prematurity (ROP) [51,52,53,54,55,56]. However, SpO_2_ alone does not provide clarity on specific end organ perfusion and NIRS may be utilised to assess real-time end organ/cerebral oxygenation status. Baerts et al. demonstrated significantly higher cerebral rSO_2_ values in preterm infants who were administered increased fractionated inspired O_2_ (FiO_2_) during an episode of desaturation, and cerebral rSO_2_ remained high for several minutes afterwards [57]. They speculate that this is may be the result of an adaptation phenomenon of post hypoxic reperfusion in a cohort of infants with limited cerebral autoregulation. Figure 5, Figure 6 and Figure 7 demonstrate fluctuations in cerebral rSO_2_ values associated with alterations in peripheral SpO_2_ values. Low cerebral rSO_2_ values are resolved by improving pulmonary oxygen uptake, by either increasing the amount of supplementary oxygen administered or increasing the mean airway pressure. Following oxygen supplementation for an episode of desaturation the rSO_2_ levels remain high, consistent with the above mentioned findings of Bearts et al. Increasing FiO_2_ was the most common intervention identified in the recent SafeBoosC intervention trial, which reported a significant reduction in the burden of cerebral hypoxia in the experimental group with cNIRS monitoring [3]. The opposite is true with cerebral hyperoxia in which high levels of supplementary oxygen administered can result in undesired elevated cerebral rSO_2_ levels, but there was no excess cerebral hyperoxia identified in the SafeBoosC trial. Isolated bradycardias have a lower impact on cerebral saturations than isolated desaturations or combined desaturation with bradycardia [58,59]. SGA infants appear to have higher cerebral rSO_2_ values along with higher haemocrit levels [60]. This is possibly related to chronic in-utero hypoxia and a redistribution of blood flow in-utero [61,62]. Studies have shown that if foetal Dopplers show evidence of brain sparing in relation to the cerebral blood flow in the foetal circulation, this effect persists in the first 72 h of life, with a relatively greater cerebral blood flow compared to renal blood flow [61].

## 5. Carbon Dioxide: Hypocarbia versus Hypercarbia

**Figure 8:**
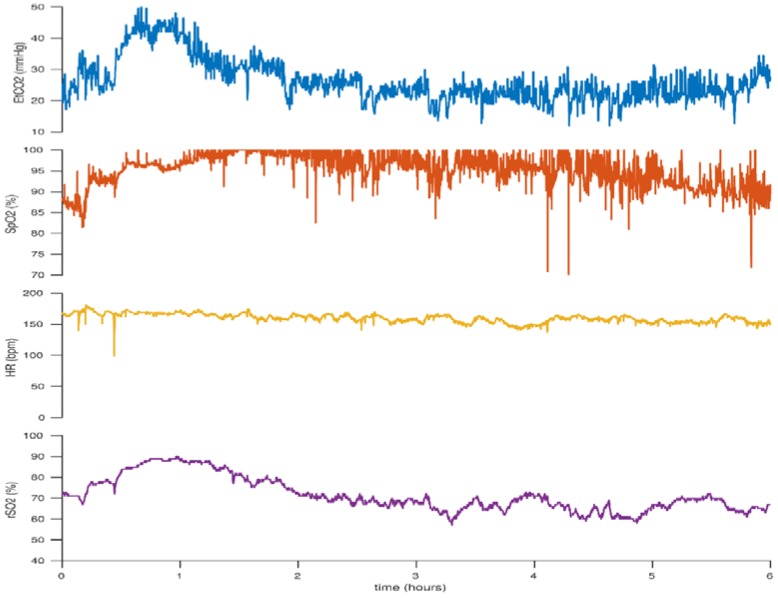
Elevated cerebral rSO_2_ values associated with increased CO_2_ levels which reduce following a decrease in CO_2_ levels. This figure reflects the case of a neonate born at 25 + 2 weeks gestation. BW 830 g, DOL 1.

Carbon dioxide is an important regulator of cerebral blood flow. Hypocapnia, particularly levels lower than 30mmHg, can negatively affect cerebral blood flow as a result of cerebral vasoconstriction [63]. This in turn leads to a reduction in oxygen delivery and can be identified by a low cerebral rSO_2_ level [64,65,66,67]. Many studies document that low PaCO_2_ levels are an important risk factor for white matter injury and subsequent development of cerebral palsy [63]. This is of particular relevance for ventilated infants where overventilation may result in hypocapnia. Low cerebral rSO_2_ may be a visual marker to prompt assessment of ventilator settings and PaCO_2_ levels. Figure 8 demonstrates a downward trend in cerebral rSO_2_ with associated low PaCO_2_ levels due to overventilation.

**Figure 9:**
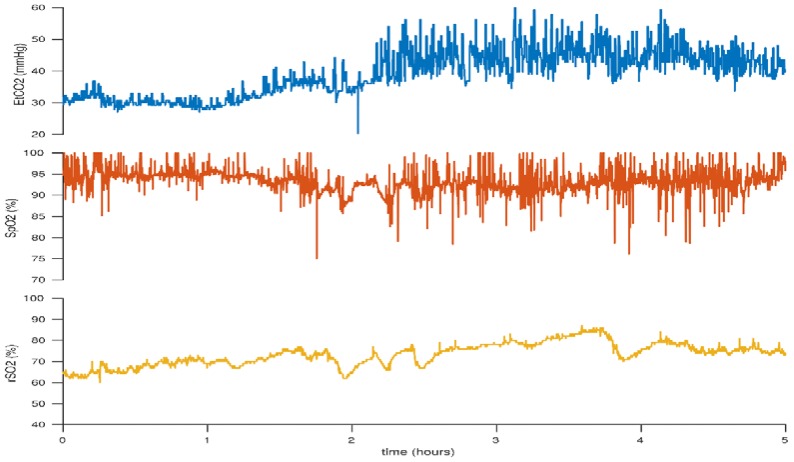
Initially low cerebral rSO_2_ and end-tidal CO_2_ (EtCO_2_) levels which increase with increasing CO_2_. This figure reflects the case of a neonate born at 23 + 6 weeks gestation. BW 530 g, DOL 2.

Conversely, the opposite is true of elevated PaCO_2_ levels. Cerebral vasodilation may lead to increased cerebral blood flow with increased oxygen delivery and cerebral hyperperfusion. Hyperoxia as a result of increased PaCO_2_ levels has also been associated with decreased brain activity as measured with electroencephalography (EEG) [68]. Cerebrovascular autoregulation is also challenged during hypercarbia [69,70,71]. Figure 9 illustrates increased cerebral rSO_2_ levels in association with increasing CO_2_ levels.

## 6. Anaemia: Anaemia versus Polycythaemia

Optimal cerebral oxygenation relies on appropriate oxygen delivery to the brain. Infants with anaemia have been shown to have lower cerebral rSO_2_ values and higher fractional tissue oxygen extraction (FTOE) than infants with normal haemoglobin levels [72]. These values normalise following blood transfusion [64,73,74,75,76]. Infants with a low cerebral rSO_2_ level and low haemoglobin level may benefit from a red cell transfusion to improve the oxygen-carrying capacity of the blood [77].

Interestingly, polycythaemia also results in impaired cerebral haemodynamics, specifically a lower cerebral blood flow velocity [78,79,80]. Partial exchange transfusions result in increased cerebral oxygenation levels likely secondary to improved cerebral blood flow [81].

## 7. Blood Glucose Level: Hypoglycaemia

Low birth weight infants are at a significantly higher risk of hypoglycaemia than their term counterparts; however, hypoglycaemia can be difficult to identify as most preterm infants are rarely symptomatic [82,83]. Hypoglycaemia is an independent risk factor for poor neurodevelopmental outcome [84,85], and thus clinicians must have a high index of suspicion and actively monitor for hypoglycaemia. cNIRS may have a role to play in hypoglycaemia monitoring in the future. Studies have shown that glycaemia affects cerebral oxygenation, particularly in the first days of life [86,87]. Low blood glucose level (BGL) is associated with increased cerebral blood flow and increased cerebral rSO_2_ levels [88]. cNIRS may be a useful indicator of low blood glucose values and its subsequent management.

## 8. Discussion

This overview highlights the various causes for fluctuations in cerebral oxygenation in preterm infants, or especially low or high absolute values, and suggests a novel individualized approach to the preterm infant. It will require a new way of thinking, where multiple parameters are given due consideration in order to establish a composite assessment of an individual infant’s current physiological status. Instead of one solution for an abnormal value, such as increasing FiO_2_ when SpO_2_ is low, one now must give careful consideration to all the potential causes for the value observed. Once the cause has been determined, a dynamic and patient specific management plan may be initiated. This multi-step and individualized way of approaching a problem may potentially reduce the infant’s burden of hypoxia/hyperoxia and the number of unnecessary interventions during the first days of life, and ultimately improve short term outcomes.

The question that must be answered before implementing such an approach is whether there is any true and relevant benefit for the patient. Does it really help to incorporate cerebral NIRS measurement in daily clinical neonatal care? The SafeBoosC trial was the first attempt at evaluating this important question. In this multicentre study, 166 preterm infants were randomly assigned to having visible cerebral NIRS measurements in combination with a pathophysiologically-oriented, evidence-based treatment guideline [89] which aimed to maintain cerebral saturation between 55 and 85%, or to the non-visible NIRS measurement and standard care. This treatment guideline contained the same items discussed in this review, for which the level of evidence varies between high quality evidence, i.e., with regards to blood transfusion for anaemia, and low-level evidence with regards to decreasing minute ventilation for pCO_2_ reduction. The cut-off values were experience-based, and the INVOS device with the adult sensor was used [90] in this particular trial. A fluctuation from baseline cNIRS measurements, as we have proposed as a prompt to assess for causation and consideration of therapeutic intervention, was not part of the original trial. Following completion of this phase 2 trial, it was clear that it is feasible to reduce the burden of cerebral hypoxia, whereas the burden of hyperoxia was not reduced in the treatment arm. Increasing FiO_2_, which is arguably the easiest intervention to increase a low cerebral rSO_2_ value, was the most common intervention used (72.1%). Other interventions included altering ventilator settings in 13.7% and commencing an inotrope/vasopressor in 5.1%. A PDA was treated as a response to low cerebral saturations in 0.4% of cases. The causes for high cerebral oxygenation are less amenable to intervention and bar lowering the FiO_2_, it is difficult to influence high rSO_2_ values other than ensuring that the PaCO_2_ value is stable.

Although cerebral hypoxia was reduced in this study, only a limited effect on the short term neurological outcomes was demonstrated. EEG and blood-derived markers for brain injury were not different between the groups [3]. Also, cranial ultrasound and brain MRI findings were not significantly different between intervention and control groups [91]. Interestingly, analysing the results as a function of burden of hypoxia, regardless of allocation to intervention or control group, the authors found more severe IVH, lower EEG burst rates and death in the infants in the fourth quartile of the burden of hypoxia, compared with the first to third quartiles. This effect was not seen for blood biomarkers [92]. We await the long-term outcome data from this group.

The fact that no short-term neurological differences were found between the experimental and control groups may potentially be due to a non-individualised approach to abnormal values; the exact and specific cause of the cerebral hypoxia may not have been targeted in each case. A ‘one size fits all’ approach of increasing FiO_2_ for all low cerebral oxygen saturation values may not result in improved outcomes as the patient may have required an alternative intervention. Improved focus on eliminating other causes for reduced oxygen supply to the brain, such as hypocarbia, might prove effective in maintaining an adequate cerebral blood flow and accompanying oxygen saturation. Although not identified in the SafeBoosC trial, ubiquitous use of increased FiO_2_ might even lead to unintended hyperoxia [57].

## 9. Conclusions

The patterns described in this review are examples of alterations in cerebral oxygenation related to differing clinical scenarios. It is hoped that these visual descriptions will assist in pattern recognition at the bedside, and will provide additional information to the clinician to guide more individualised interventions. The ultimate usefulness of cNIRS will need to be evaluated in a larger clinical trial. A larger phase 3 SafeBoosC trial is being planned which aims to enroll 1500 preterm infants to examine if cerebral NIRS can reduce the risk of death or severe brain injury at 36 weeks of gestation [93] This study aims to assess if monitoring and treating episodes of cerebral hypoxia and hyperoxia, results in improved outcomes for preterm infants. In the meantime, cNIRS is currently providing many valuable insights into the cerebral haemodynamics and effects of therapeutic interventions in this very vulnerable population. This tool requires further study in order to fully ascertain its role in active neonatal management and ability to prognosticate neurodevelopmental outcome. It is hoped that the patterns described in this review will assist in identification of potential causes of altered cerebral oxygen saturation, thus providing the clinician with additional information to make a more informed management choice.
